# Evaluation of copromicroscopy and serology to measure the exposure to *Ascaris* infections across age groups and to assess the impact of 3 years of biannual mass drug administration in Jimma Town, Ethiopia

**DOI:** 10.1371/journal.pntd.0008037

**Published:** 2020-04-13

**Authors:** Daniel Dana, Johnny Vlaminck, Mio Ayana, Bamlaku Tadege, Zeleke Mekonnen, Peter Geldhof, Bruno Levecke

**Affiliations:** 1 School of Laboratory Science, Faculty of Health Science, Institute of Health, Jimma University, Jimma, Ethiopia; 2 Department of Virology, Parasitology and Immunology, Ghent University, Merelbeke, Belgium; 3 School of Medicine, Hawassa University, Hawassa, Ethiopia; University of Calgary, CANADA

## Abstract

**Background:**

The scientific community has recently summarized the desired characteristics for diagnostic tools across the different phases of a soil-transmitted helminth (STH) mass drug administration (MDA) program. Although serology meets some of the desired criteria, there is a scarcity of data on baseline serological profiles in human populations, both prior to and during MDA programs.

**Methods:**

In this study, we compared the copromicroscopic and the serological infection profiles in 600 school-aged children (SAC) and 600 adults at the advent of the MDA program in Jimma Town, Ethiopia. The serological profiles were examined by two ELISAs that measure IgG4 responses to the *Ascaris suum* haemoglobin antigen (AsHb) and a somatic extract of lung stage larvae (AsLungL3). Three years into the MDA program, we sampled another group of 600 SAC from the same schools to assess the reduction in prevalence and intensity of *Ascaris* infections measured by copromicroscopy and serology.

**Principal findings:**

Prior to the start of MDA, copromicroscopy revealed an *Ascaris* prevalence of 31.0% and a mean fecal egg count of 2,919 eggs per gram (EPG) in SAC. Following three years of biannual treatment, the prevalence reduced to 13.2% (57.8% reduction) and the mean fecal egg count to 1,513 EPG (48.1% reduction). This reduction was also reflected in the serological results. The seroprevalence reduced with 40.9% and 27.4% and the mean optical density ratio reduced with 44.2% and 38.2% as measured by the AsHb or AsLungL3 ELISA respectively. We also showed that, despite a decreasing coproprevalence, seroprevalence to *Ascaris* increased with age.

**Conclusions:**

This study is the first to provide IgG4 response profiles of an endemic population to two different *A*. *suum* antigens. The results suggest that exposure to the infectious stages of *Ascaris* reaches beyond SAC alone. Furthermore, it highlights the possible use of serological assays to monitor changes in STH exposure during MDA programs.

## Author summary

Worldwide, there has been an upscale in deworming programs to reduce the disease burden attributable to intestinal worms (giant roundworms, whipworms and hookworms). As a consequence of this, there is a focus-shift from morbidity control towards breaking transmission and ultimately disease elimination. Current standard diagnostic tools, which are based on the demonstration and quantification of worm eggs in stool (copromicroscopy), lack the diagnostic performance to make well-founded decisions when a program reaches its endgame. The scientific community has therefore defined minimal criteria that new potential diagnostic methods should meet. Diagnostic tools that are based on the detection of antibodies, which are indicative of the natural immune response of the host to worm-specific components in blood, potentially meet these criteria. We compared giant roundworm infection profiles by copromicroscopy and two antibody-based tests in both children and adults in an endemic setting. Three years into the national deworming program, another group of children from the same schools were sampled to evaluate the program progress. Our results provide a first step in the evaluation of antibodies as a diagnostic tool in the endgame of deworming programs, but they also highlight the need for further research on the interpretation of antibody-based diagnostics for STH.

## Introduction

In 2012, more than 70 pharmaceutical companies, governments, and global health organizations signed the London Declaration on Neglected Tropical Diseases (NTDs) and committed themselves to control and eliminate NTDs at the individual patient level or at the population level through programs of mass drug administration (MDA). Soil-transmitted helminthiasis is caused by a group of intestinal worms (*Ascaris lumbricoides*, *Trichuris trichiura* and two hookworm species (*Ancylostoma duodenale* and *Necator americanus*)) and is considered one of the most important NTDs that is amenable to control through MDA. It has been estimated that these worms affect 1.45 billion people, which accounts for 11% of the global burden attributable to NTDs [1, 2]. So far, major progress has been made in combating soil-transmitted helminthiasis, significantly reducing its global disease burden [3]. This, together with the prospect of sustained anthelmintic drug donations, has caused a focus-shift from morbidity control towards breaking transmission and ultimately disease elimination [4, 5].

Disease elimination programs go through a variety of phases, and programmatic decisions to transition from one program phase to the next are driven by the outcome of large epidemiological surveys applying standard diagnostic tools [6–8]. Currently, the microscopic detection and quantification of eggs excreted by adult worms in stool remains the standard diagnostic tool [9]. However, copromicroscopy lacks the performance to make well-founded decisions when a program reaches its endgame [10, 11]. In fact, implementing these methods may potentially lead to substantial health and monetary consequences resulting from a premature stop decision.

As a response to this urgent need for improved diagnostic tools, the community summarized the desired characteristics–the so called target product profiles—for the different phases of the program (use-case #1: determine disease transmission and identify type of MDA, use-case #2: assess progress against program goals, use-case #3: confirm a decision to stop intervention and transition into surveillance and use-case #4: verify a sustained break in transmission; [10]). For the last two use-cases, the community concluded that the desired diagnostic biomarker should be in sufficient abundance in a readily accessible body fluid, excluding stool. Potential biomarkers that meet this desired characteristic are infection-induced host antibodies. Measuring the presence and concentration of these antibodies, as a proxy for parasite exposure, has already been proven helpful in the elimination program of lymphatic filariasis, another MDA amenable NTD [12].

Recent studies have proven the usefulness of serological assays for *Ascaris suum* diagnosis in pigs. Serum antibody responses against an antigen from adult worms (*A*. *suum* haemoglobin (AsHb) and the total extract of *A*. *suum* lung stage L3 larvae (AsLungL3) showed higher sensitivity than copromicroscopy to identify exposure to both experimental and natural *A*. *suum* infections [13, 14] and provided more accurate assessments of disease burden [14–18]. It is however difficult, if not impossible, to obtain similar high-quality data from studies in human populations. Nevertheless, a recent study showed a significant reduction in serum antibody response to AsHb in an Indonesian human population during the course of a community-based MDA control program targeting lymphatic filariasis and soil-transmitted helminthiasis, already highlighting the potential of serology to assess progress against program goals in STH control programs (use case #2) [19].However, today, there is a scarcity of data on baseline serological profiles to these antigens in human populations of endemic and non-endemic areas. This limits our insights into biomarker-specific infection profiles and impedes the evaluation of the clinical utility of serology in the different phases of a MDA program.

Therefore, the main aims of this study were (i) to assess diagnostic cutoffs for both AsHb and AsLungL3 using sera from a non-endemic population, (ii) to compare the serological profiles of school-aged children (SAC) and adults from an endemic population in Ethiopia with infection profiles obtained by copromicroscopy and (iii) to evaluate the impact of three years of bi-annual MDA on STH infections applying copromicroscopy and *Ascaris* serology as a proof of concept.

## Methods

### Ethical considerations

Blood samples from the non-endemic adult population in Belgium were acquired through Red Cross Flanders (order numbers: CG2016 0404A, CM2016 0627B and CG2016 1219F). For the collection of stool and blood samples in Jimma, Ethiopia, ethical approval was obtained from the Institutional Review Board of both Ghent University, Belgium (reference number: 2015/0801 and study registration number: B670201526293) and Jimma University, Ethiopia (reference number: RPGC/181 and IHRPGD/680). For SAC, the school authorities, teachers, parents, and the children were informed about the purpose and procedures of the study. A written informed consent was obtained from the parents/guardians. An additional separate written consent was secured from children older than 12 years. Consent forms were prepared in English, translated into the two commonly used local languages (Afaan Oromo and Amharic) and a copy was handed over to the children’s parents/guardians. Only those children who were willing to participate and whose parents or guardians signed the written informed consent form were included in the study. For the adult population, we only involved subjects in the study that provided their signed informed consent before data collection. All individuals infected with any STH based on copromicroscopy were administered a single oral dose of 400 mg albendazole (GlaxoSmithKline).

### Study design

Three cross-sectional surveys were performed in Jimma Town, Southwest Ethiopia. In a first survey (October–December 2015), we screened 600 SAC from ten primary schools across 6 *kebeles* (local language for neighborhoods). The selection of the primary schools was based on the presence of the age groups of interest (5 to 18 years) and their participation in previous studies [20–22]. In each school, 60 subjects were sampled (30 children between the age 5 and 10, and 30 children between the age of 14 and 18), resulting in a total of 600 SAC. In a second survey (February to May 2017), we screened 600 adults across the 6 *kebeles* (100 per *kebele*). After three years of bi-annual school-based MDA, a third survey was conducted from November to December 2018. This survey included another sample of 600 randomly selected SAC from the same ten primary schools that were included during the first survey. An overview of the number of SAC and adults sampled per *kebele* is provided in **S1 Table**.

Individuals consenting to participate in the study were asked to provide at least 5 g of fresh stool in a clean and labeled stool container. Because we wanted to maximize the clinical sensitivity of our analysis and produce the most accurate infection profiles (minimizing the number of false negative cases) in survey 1 (SAC) and 2 (adults), we processed all stool samples by Kato-Katz (0.0417 g of stool), McMaster egg counting method (2 g of stool) and Mini-FLOTAC (2 g of stool) for the detection and enumeration of STH eggs. Each of these methods has been validated for diagnosis of STH infections [23]. In the third survey (SAC), stool samples were only examined by Kato-Katz, as this is the current recommended diagnostic method to evaluated progress made in MDA programs for STH. In addition to the stool sample, blood was collected from each individual. Blood samples were centrifuged for 10 min at 4,000 g and 4˚C. Subsequently, the serum was recovered and stored at -20˚C. All sera were shipped on dry ice to the Laboratory of Parasitology at Ghent University (Belgium) for serological analysis.

### Copromicroscopy

#### Kato-Katz

Kato-Katz thick smears (Kato-Katz) were prepared per individual as described by WHO [9, 24]. To avoid clearance of hookworm eggs, all smears were examined within 30–60 min for the presence of STH eggs. The number of STH eggs was counted as per species basis and multiplied by 24 to obtain the fecal egg counts (FECs; expressed in eggs per gram of stool (EPG)).

#### McMaster

The McMaster egg counting method was performed as previously described [25]. In short, 2 g of stool was suspended in 30 ml of flotation solution (saturated salt solution at room temperature with specific density ~1.20). The stool suspension was poured three times through a wire mesh to remove large debris. Then, aliquots of 0.5 ml were transferred to each of the two chambers of a McMaster slide. Finally, the numbers of STH eggs were counted in each of the two chambers using light microscopy at a 100x magnification. The FECs were obtained by multiplying the total number of eggs by 50 for each of the STH species.

#### Mini-FLOTAC

The Mini-FLOTAC was performed as described by Cringoli and colleagues [24, 26, 27]. In short, 2 g of fresh stool was weighed into the Fill-FLOTAC container, followed by addition of 38 ml of flotation solution (same as for McMaster). Then the stool was thoroughly homogenized using the homogenizer stick of the Fill-FLOTAC. The fecal suspension was filtered through the Fill-FLOTAC and subsequently added to both chambers of the Mini-FLOTAC device. After 10 minutes, the top part of flotation chambers was translated and the numbers of STH eggs were counted in each of the two chambers using light microscope at a 100x magnification. The FECs were obtained by multiplying the total number of eggs by 10 for each of the STH species.

#### Serology

Serological analysis of the serum samples was performed based on the protocols described by Vlaminck *et al*. for the AsHb ELISA [19] and Vandekerckhove *et al*. for the AsLungL3 ELISA [13]. These ELISAs are designed to measure IgG4 responses as it has been shown by Santra et al., 2001 [28] and Chatterjee et al., 1996 [29] that human IgG4 responses to *Ascaris* antigens showed less cross reactivity than IgG1, IgG2 and IgG3 subclass antibodies in sera from patients infected with hookworm, *Trichuris* and *Strongyloides*.

Maxisorp 96-well ELISA plates were coated overnight at 4˚C with 1μg/ml AsHb and 5μg/ml of AsL3Lung in carbonate buffer (pH = 9.6). After three wash steps with the wash buffer (PBST: phosphate buffered saline containing 0.05% Tween20), non-specific binding sites were blocked for 1 hour at room temperature using blocking buffer (PBS + 5% heat treated fetal calf serum). The coated antigens were detected by adding diluted sera and controls (positive—(PC) and negative controls (NC)) at a 1:200 dilution for AsHb and 1:400 dilution for AsLungL3 in PBST for 2 hours at room temperature. Positive and negative controls were added to each plate. All samples were run in duplicate. Following three subsequent wash steps, horseradish peroxidase conjugated anti-human IgG4 (1: 20,000 dilution) in blocking buffer was added to the plate for 1 hour at room temperature to detect IgG4 antibodies that were bound to the antigens. After a final three washing steps with PBST, 100 μl of substrate was added (O-phenylenediamine 0.1% in citrate buffer (pH = 5.0)). The development reaction was stopped after 10 min with 2.5M H_2_SO_4_ and the optical density (OD) measured at 492 nm. Reactivity to the AsHb and AsLungL3 antigen is shown in optical density ratio (ODr = (OD_sample_−OD_NC_)/(OD_PC_−OD_NC_)). A collection of 500 blood samples from healthy Belgian blood donors were also screened to determine the diagnostic cutoff (positive *vs*. negative test result). The cutoff was established as mean ODr ± 3x standard deviation.

To exclude potential cross-reactivity with *Toxocara spp*, a subset of blood samples from healthy Belgian donors was evaluated for antibodies against *Toxocara* L3 excretory/secretory proteins using a commercial IgG ELISA kit (Cypress diagnostics, Belgium). This ELISA was performed according to the manufacturer’s instructions. Samples were classified as positive (ODr >1.1), negative (ODr <0.9) or equivocal (1.1≥ ODr ≥0.9).

#### Statistical analysis

For surveys 1 and 2, the detection of patent STH infections (coproprevalence) was based on the combined test result of single Kato-Katz, McMaster and Mini-FLOTAC. An individual was considered to be infected with a particular STH species, when at least one egg was found in his/her stool sample using any of the three copro-microscopic methods. The intensity of the infections was estimated by taking the average FEC over the three copro-microscopic methods (surveys 1 and 2). The detection of exposure to *Ascaris* infection as measured by the IgG4 antibody response to parasite antigens (AsHb and AsLungL3) was determined as the percentage of individuals that tested positive on the serological assay (test-ODr > diagnostic cutoff) (seroprevalence). To be able to calculate the reduction in mean ODr measured by both assays that can be compared to the reduction in mean FECs, all negative ODr values (i.e. samples with OD values below that of the negative reference sample) were given a zero value.

Statistically significant differences in qualitative (positive and negative test result) *Ascaris* test results were assessed using the ‘glmer’ function in R. The test-result (positive or negative) was used as dependent variable while the parameters: ‘test’ (3 levels: copromicroscopy, AsLungL3 and AsHb), ‘kebele’ (9 levels: Awetu, Mendera, Bacho Bore, Bossa Addis, Bossa Kitto, Ginjo, Hermata, Jiren, Mentina, Seto Semaro), ‘sex’ (2 levels: male and female) and ‘age of subject’ (in years) were included as potential predicting variables. For a simplified interpretation of the effect of age, the age was centralized around the median age (18 years).

In addition, we assessed the reduction in both prevalence and intensity of STH infections. For copromicroscopy, the infection intensity was measured as the mean FECs and the proportion of low, moderate-to-heavy intensity infections as defined by WHO [30]. For serology, the intensity of exposure was measured as the mean ODr obtained by ELISA. The 95% confidence intervals (95% CI) around the point estimates were determined through bootstrap analysis (5,000 iterations) for all infection parameters. We concluded that reduction in prevalence or intensity was significant when the 95% CI interval did not include zero. Finally, correlations between ODr and mean FEC were determined using the Pearson correlation coefficient. The level of significance was set at *p*<0.05.

## Results

### Study population

A total of 1,800 participants were sampled during the 3 surveys performed in this study. Namely, 1,200 SAC from 10 primary schools (600 in 2015 and 600 in 2018) and 600 adults from the neighborhoods (*kebeles*) where the schools were located. All of the participants provided sufficient stool to perform all copro-microscopic methods and a sufficient amount of blood to perform serology.

### STH infection profiles in the population prior to MDA

The cross-sectional surveys of SAC and adults at the start of the MDA program resulted in a total study population of 704 female and 496 male individuals across 9 *kebeles* (Awetu Mendera: 60; Bacho Bore: 160; Bossa Addis: 160; Bossa Kitto: 160; Ginjo: 220; Hermata: 60; Jiren: 60; Mentina: 160; Seto Semaro: 160) and 5 age groups (age 5 to 10: 300; age 14 to 17: 263; age 18 to 29: 310; age 30 to 49: 208; at least 50 years of age: 119).

**Fig 1:**
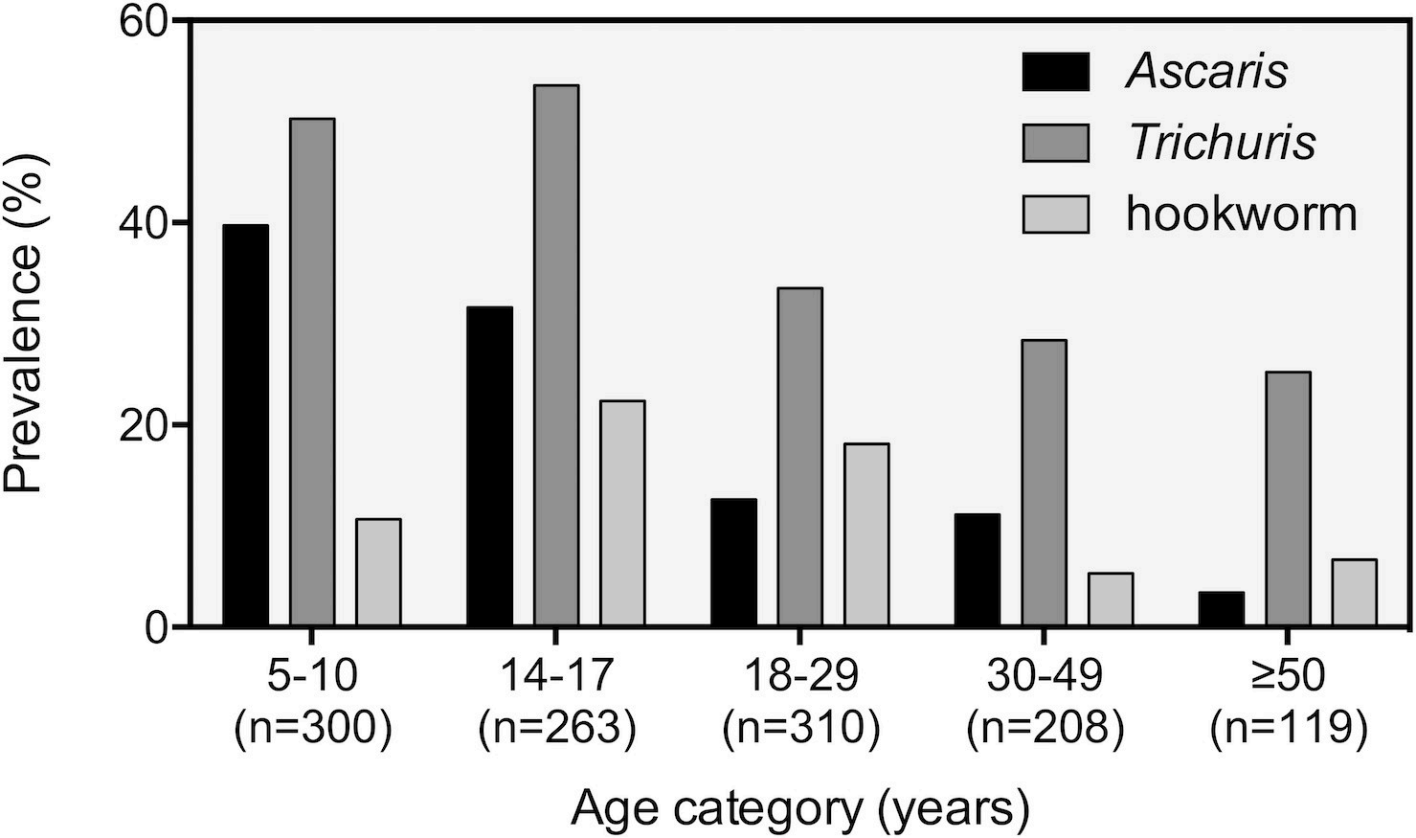
Coproprevalence of soil-transmitted helminth infections across five age groups in Jimma Town, Ethiopia.

The goal of the first two surveys was to get an overview of the spread and intensity of STH infections in the whole community in Jimma Town, Ethiopia. To increase diagnostic sensitivity, stool samples were analyzed using three copro-microscopic methods (Kato-Katz, McMaster and Mini-FLOTAC) and the combined result of these analysis was used to determine the prevalence of any STH. The overall presence of STH in the whole population (1,200 individuals) was 52.9% (635/1,200). *Trichuris* infections were the most prevalent (40.4%), followed by *Ascaris* (22.3%) and hookworm infections (13.8%). Males were more infected than females for each of the three STH species (*Ascaris*: 27.2% *vs*. 18.9%; *Trichuris*: 44.8% *vs*. 37.4%; hookworms: 16.7% *vs*. 11.8%). The prevalence for each STH per age group is illustrated in **Fig 1**. This revealed that the highest prevalence for *Ascaris* (39.7%) occurred in the youngest age group (age 5 to 10). *Ascaris* prevalence steadily dropped as individuals grew older, with only 3.4% of individuals older than 50 excreting *Ascaris* eggs. Across the different age groups, *Trichuris* infection was most common in teenagers of 14 to 17 years of age (53.6%). *Trichuris* prevalence also reduced with age, but it never dropped below 25%. Hookworms were the least prevalent of all STHs, with a maximum prevalence of 22.4% in SAC aged 14 to 17. Just like for the other two STH species, hookworm prevalence dropped in the adult population from 18.1% in the 18 to 29-year-olds to 5.3% in the 30 to 49-year-olds and 6.7% in the individuals of age 50 and above. Generally, the mean FEC across the five age groups and both sexes followed trends similar to that of prevalence, with the highest mean FECs observed in the age groups/sex with the highest prevalence. **S1 Table** provides the prevalence and intensity of *Ascaris*, *Trichuris* and hookworm infections across the five age groups, both sexes and nine *kebeles*.

### STH infection profiles in SAC after 3 years of bi-annual MDA

The same 10 primary schools that participated in 2015 (survey 1) were re-sampled in 2018 (survey 3) to assess the impact of three years of bi-annual MDA on the prevalence and intensity of STH infections. Stool samples collected from the 600 SAC in 2018 were only evaluated by Kato-Katz. This is because WHO recommends single Kato-Katz for monitoring the progress of STH MDA programs [30]. To compare the infection outcomes between both surveys, only Kato-Katz results were used. The copromicroscopic results of for both surveys are provided in **Table 1**.

**Table 1:** The impact of deworming on soil-transmitted helminth (STH) infections in school-aged children (SAC) measured by copromicroscopy and serology. Copromicroscopic data is expressed as the prevalence with 95% confidence interval (95 CI) of any, low or moderate-to-high (M-H) intensity infections. The mean fecal egg count (FEC) is expressed in eggs per gram (EPG) stool. Serological response to *Ascaris suum* haemoglobin (AsHb) or total lung stage L3 extract (AsLungL3) is reported as mean Optical Density ratio (ODr) as measured by ELISA.

			2015 (n = 600)	2018 (n = 600)	Reduction (%) (95% CI)
Corpromicroscropy					
Any STH	Prevalence (%) (95 CI)				
		Any intensity	57.7 (53.8; 61.7)	28.0 (24.5; 31.5)	51.5 (44.4; 58.2)
		Low intensity	45.4 (41.3; 49.3)	22.2 (18.8; 25.5)	51.1 (42.5; 59.3)
		M-H intensity	12.3 (9.8; 15.2)	5.8 (4.0; 7.8)	52.8 (31.7; 68.7)
*Ascaris*	Prevalence (%) (95 CI)				
		Any intensity	31.3 (27.7; 35.2)	13.3 (10.7; 16.2)	57.5 (46.8; 66.7)
		Low intensity	20.2 (17.0; 23.3)	8.0 (5.8; 10.3)	60.4 (46.5; 71.7)
		M-H intensity	11.2 (8.8; 13.8)	5.3 (3.7; 7.2)	52.7 (29.8; 69.2)
	Mean FEC (EPG) (95 CI)		2,919 (1,999; 4,115)	1,514 (894; 2,227)	48.1 (11.0; 71.9)
*Trichuris*	Prevalence (%) (95 CI)				
		Any intensity	39.5 (35.7; 43.3)	18.2 (15.2; 21.2)	53.9 (44.7; 62.4)
		Low intensity	37.0 (33.2; 41.0)	16.9 (14.0;19.7)	54.3 (44.8; 63.3)
		M-H intensity	2.5 (1.3; 3.8)	1.3 (0.5; 2.3)	48.0 (-25.0; 81.0)
	Mean FEC (EPG) (95 CI)		165 (117; 222)	54 (37; 73)	67.3 (49.1; 79.8)
Hookworm	Prevalence (%) (95 CI)				
		Any intensity	10.3 (8.0; 12.8)	5.0 (3.3; 6.8)	51.5 (28.8; 69.1)
		Low intensity	10.3 (8.0; 12.8)	5.0 (3.3; 6.8)	51.5 (28.8; 69.1)
		M-H intensity	0.0 (0.0; 0.0)	0.0 (0.0; 0.0)	_
	Mean FEC (EPG) (95 CI)		23 (15; 33)	10 (6; 15)	56.5 (25.3; 78.5)
Serology					
AsHb ELISA	Prevalence (%) (95% CI)		3.7 (2.3; 5.3)	2.2 (1.2; 3.3)	40.5 (-13.3; 71.9)
	Mean ODr		0.052 (0.040; 0.067)	0.029 (0.022; 0.038)	44.2 (19.5; 61.8)
AsLungL3 ELISA	Prevalence		54.2 (50.2; 58.2)	39.3 (35.5; 43.2)	27.5 (18.3; 35.8)
	Mean ODr		0.616 (0.537; 0.699)	0.380 (0.317; 0.448)	38.3 (24.0; 50.9)

In 2015, any STH infections were detected in 57.7% of sampled children, with *Trichuris* being the most prevalent (39.5%) followed by *Ascaris* (31.3%) and hookworm infections (10.3%). The prevalence of moderate-to-heavy intensity was 12.3% for any STH infections, 11.3% for *Ascaris* and 2.5% for *Trichuris*. All hookworm infections were of low intensity. The mean FEC followed a similar pattern across the STH species, the mean FEC being highest for *Ascaris* (2,919 EPG) and the lowest for hookworm infections (23 EPG). For *Trichuris*, mean FEC equaled 165 EPG. In 2018, following three years of biannual deworming, the prevalence dropped to 28.0% for any STH infection, 18.2% for *Trichuris*, 13.3% for *Ascaris* and 5.0% for hookworm infections. This corresponds to a significant (the 95% confidence interval does not zero) reduction in prevalence of 51.5% (95CI: 44.4–58.2), 57.5% (95CI: 46.8–66.7), 53.9% (95CI: 44.7–62.4) and 51.5% (95CI: 28.8–69.1), respectively. In line with these reductions in prevalence, there was also a reduction in infection intensity (measured as the reduction in prevalence of low and moderate-to-heavy intensity infections and mean FECs) for all STH species. The reduction for these parameters varied ranged from 48.0% to 67.3%, and was significant for all parameters except for *Trichuris*. For this STH, a non-significant reduction in moderate-to-heavy intensity infections was observed (48.0%, 95CI: -25.0–63.3). No moderate-to-heavy intensity hookworm infections were detected in 2018.

#### Establishment of diagnostic cutoffs of serological assays

**Fig 2:**
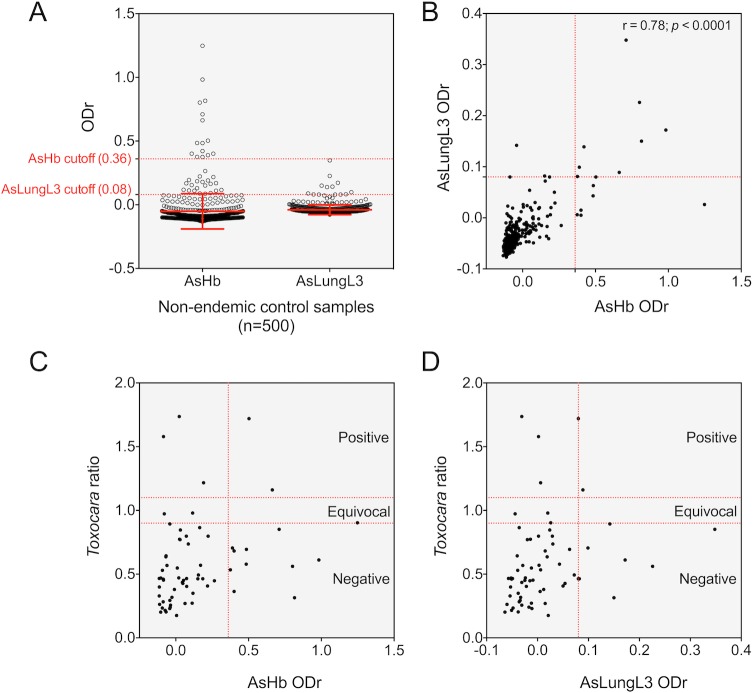
Serological screening of a non-endemic adult population from Belgium. Panel A shows the result of a serological screening of 500 healthy Belgian adults using the *Ascaris suum* haemoglobin (AsHb) and lung L3 extract (AsLungL3) ELISA. Horizontal dotted lines in red represent the calculated diagnostic cutoffs for each ELISA test (mean optical density ratio (ODr) of all samples + 3x the standard deviation). Group means and standard deviations are indicated with a red dotted line. Panel B shows the correlation between the IgG4 responses to AsHb and AsLungL3 in these samples. The Pearson correlation coefficient (r) and its respective significance level (*p*) is reported in the top right of the graph. Panel C and D illustrate the results of a commercial *Toxocara* ELISA and the AsHb or AsLungL3 ELISA on a subset of 65 Belgian adult sera. Here, the horizontal dotted lines represent the cutoffs that were applied to categorize the samples into negative (ratio <0.9), equivocal (0.9 ≤ ratio ≤1.1) or positive (ratio >1.1). The vertical dotted lines represent the test-specific cutoffs determined for the ELISAs with *Ascaris* antigens.

We first evaluated the IgG4 response to AsHb and AsLungL3 in a set of 500 healthy adult individuals from a non-endemic population (Belgium). This was done to establish a diagnostic cutoff for both serological assays and to record the serological reactivity that can be expected of a non-endemic adult population (**Fig 2A**). The cutoffs for both tests were determined by adding 3 times the standard deviation to the mean ODr obtained by each test. For the AsHb ELISA, this resulted in a cutoff ODr of 0.36 while the cut-off ODr for the AsLungL3 ELISA positivity was set at 0.08. The results of both ELISAs were significantly correlated (Pearson’s coefficient = 0.78; *p* <0.0001) (**Fig 2B**).

Remarkably, a number of Belgian individuals showed significant reactivity to both antigens. To investigate if the reactivity to *Ascaris* antigens in these individuals was caused by exposure to *Toxocara* species (a zoonotic helminth species that is prevalent in dogs and cats in Belgium [31, 32]), their response to *Toxocara* larval excretory/secretory antigens was analyzed by a commercial ELISA. For this, we selected 65 individuals, including 35 individuals who showed elevated IgG4 responses to AsHb (ODr >0.10) and AsLungL3 (ODr >0.00) and 30 randomly selected individuals who did not show any response to either *Ascaris* antigen (ODr <0.00). Of these 65 examined Belgian serum samples, 5 were positive on the *Toxocara* ELISA (**Fig 2C and 2D**). Only two out of 13 and one out of eight samples that were positive for the AsHb or AsLungL3 ELISA respectively had a positive signal on the *Toxocara* ELISA. Apart from three samples with an equivocal result, all remaining samples tested negative on the *Toxocara* ELISA.

#### Anti-*Ascaris* IgG4 profiles of endemic population prior to MDA

**Fig 3:**
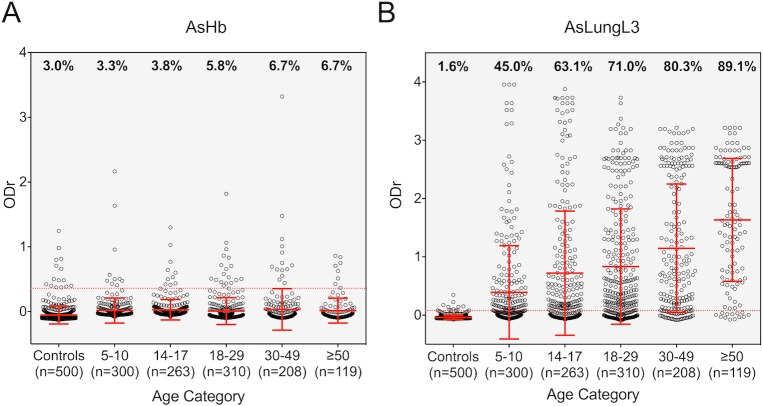
IgG4 responses to AsHb and AsLungL3 by age. The serological IgG4 response to *Ascaris suum* haemoglobin (AsHb) (**panel A**) and lung L3 extract (AsLungL3) (**panel B**) in 500 non-endemic Belgian adults (controls) and 1,200 individuals (600 school-aged children from survey 1 and 600 adults from survey 2) from Jimma Town (Ethiopia). Individuals from the endemic area are categorized by age. Group means and standard deviations are indicated with a full red line. Horizontal red dotted lines indicate the test-specific cutoffs. The percentage of individuals crossing the test cutoff is mentioned on top of each age group.

Seroprevalence varied highly depending on the test used, with 66.2% of the population testing positive on the AsLungL3 ELISA and 5.0% testing positive on the AsHb ELISA. Compared to the copromicroscopy, two important differences were observed. First, in contrast to the age specific infection profile for copromicroscopy, for which *Ascaris* prevalence decreased as a function of increasing age, there was an increase in seroprevalence as a function of increasing age for both ELISAs (**Fig 3**). Seroprevalence measured by AsHb ELISA increased from 3.3% in the youngest age group to 6.7% in the oldest age group. Using the AsLungL3 ELISA, seroprevalence in the youngest age group was 45.0% and also reached a maximum in the adults older than 50. In this age group, nearly all individuals had seroconverted (89.1%). Second, although a difference in coproprevalence was observed between males (27.2%) and females (18.9%), this difference was less pronounced when applying serology (AsHb: 5.6% *vs*. 4.5%; AsLungL3: 66.5% *vs*. 65.9%). **S2 Table** provides the seroprevalence and mean IgG4 response to *Ascaris* antigens, based on the two ELISAs across the five age groups, both sexes and nine *kebeles*.

The outcomes of the generalized mixed models confirmed the significant differences in the test result between the three tests (copromicroscopy, AsHb and AsLungL3 EILSA) and the impact of age, but not sex or *kebele*. At an age of 18 (reference age = median age) and compared to copromicroscopy (reference test), the odds of testing positive on ELISA is 6.0 times higher (95CI: 4.80–7.48) using the AsLungL3 antigen and 6.86 times lower (95CI: 4.93–9.54) using the AsHb ELISA. The odds for a positive copro-microscopic test result decreased significantly with increasing age (OR: 0.94; 95CI: 0.92–0.96). For both ELISAs, this change in odds in a positive test result over age was significantly different. Compared to copromicroscopy, there was a net increase in odds ratio with a factor 1.08 (95CI: 1.06–1.10) and 1.12 (95CI: 1.10–1.14) per 1-year increase from the reference age for AsHb and AsLungL3 respectively. No significant differences in test results across both sexes and *kebeles* were observed.

#### Impact of 3 years of bi-annual MDA on anti-Ascaris IgG4 antibody levels in SAC

In the year 2015, just prior to the start of MDA, 3.7% and 54.2% of the SAC in Jimma had tested positive on the AsHb and AsLungL3 ELISA, respectively. In 2018, after three years of bi-annual deworming we observed a seroprevalence of 2.2% on the AsHb ELISA, corresponding to a non-significant (the 95 CI includes zero) reduction in seroprevalence of 40.5% (95% CI: -13.3–71.9). The seroprevalence measured by the AsLungL3 ELISA dropped to 39.3% resulting in a significant reduction of 27.5% (95% CI: 18.3; 35.8) (**Table 1**).

**Fig 4:**
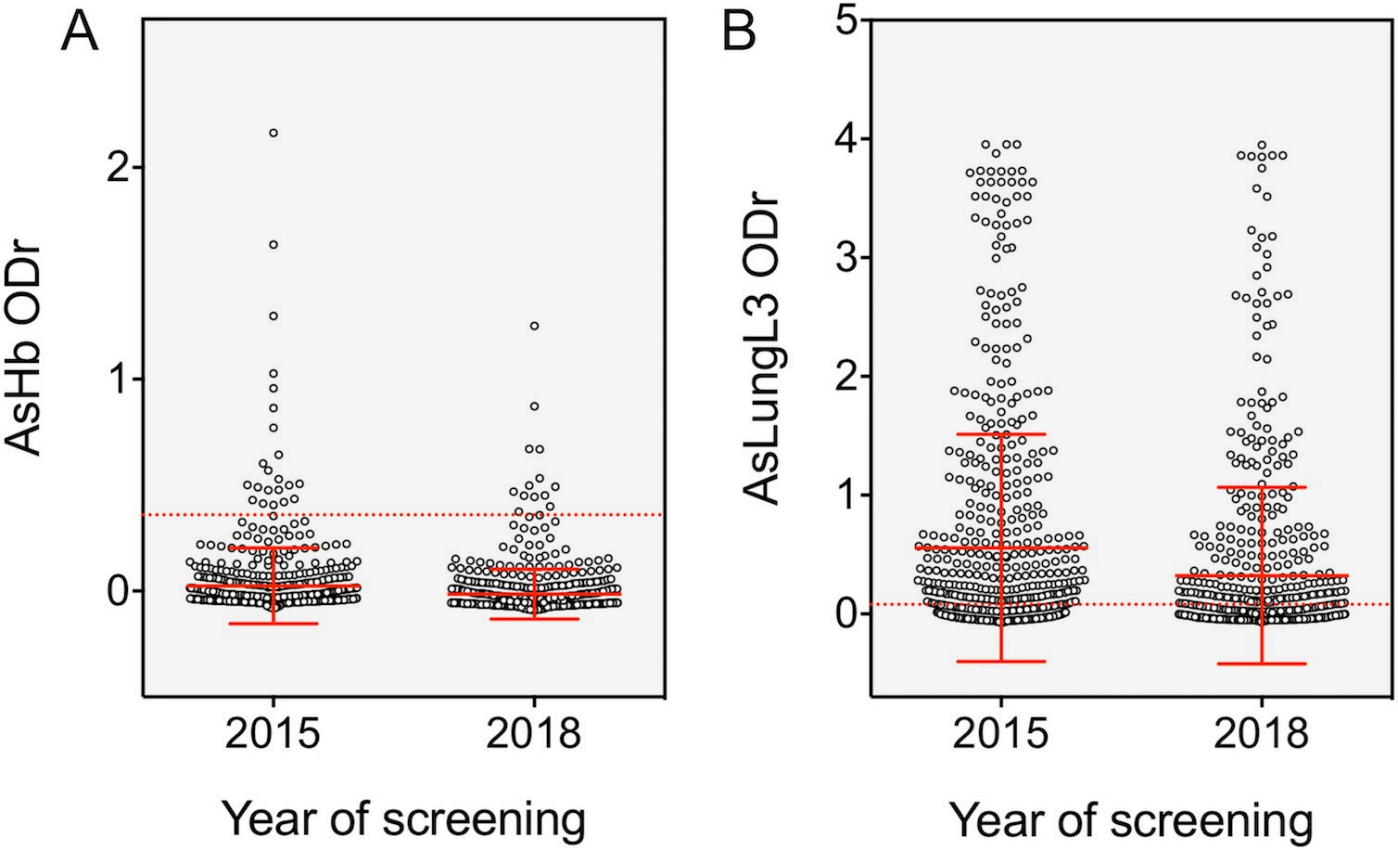
IgG4 responses to AsHb and AsLungL3 before and three years into the deworming program. The IgG4 responses to *Acaris suum* haemoglobin (AsHb) (A) and lung L3 extract (AsLungL3) (B) measured in 600 school-aged children from Jimma, Ethiopia, before (2015) and after (2018) 3 years of biannual mass drug administration with mebendazole. Horizontal red dotted lines represent the calculated diagnostic cutoffs for each ELISA test. Group averages and their standard deviation is indicated by full red lines.

In 2015, the mean ODr measured in the 600 SAC by the AsHb and AsLungL3 ELISA, was 0.052 and 0.616 respectively. In 2018, this had reduced significantly to a mean ODr of 0.029 for the AsHb ELISA (reduction of 44.2%; 95% CI: 19.5–61.8) and an ODr of 0.380 for the AsLungL3 ELISA (reduction of 38.3%; 95% CI: 24.0–50.9) (**Fig 4)**.

### Agreement between FECs and ODr

**Fig 5:**
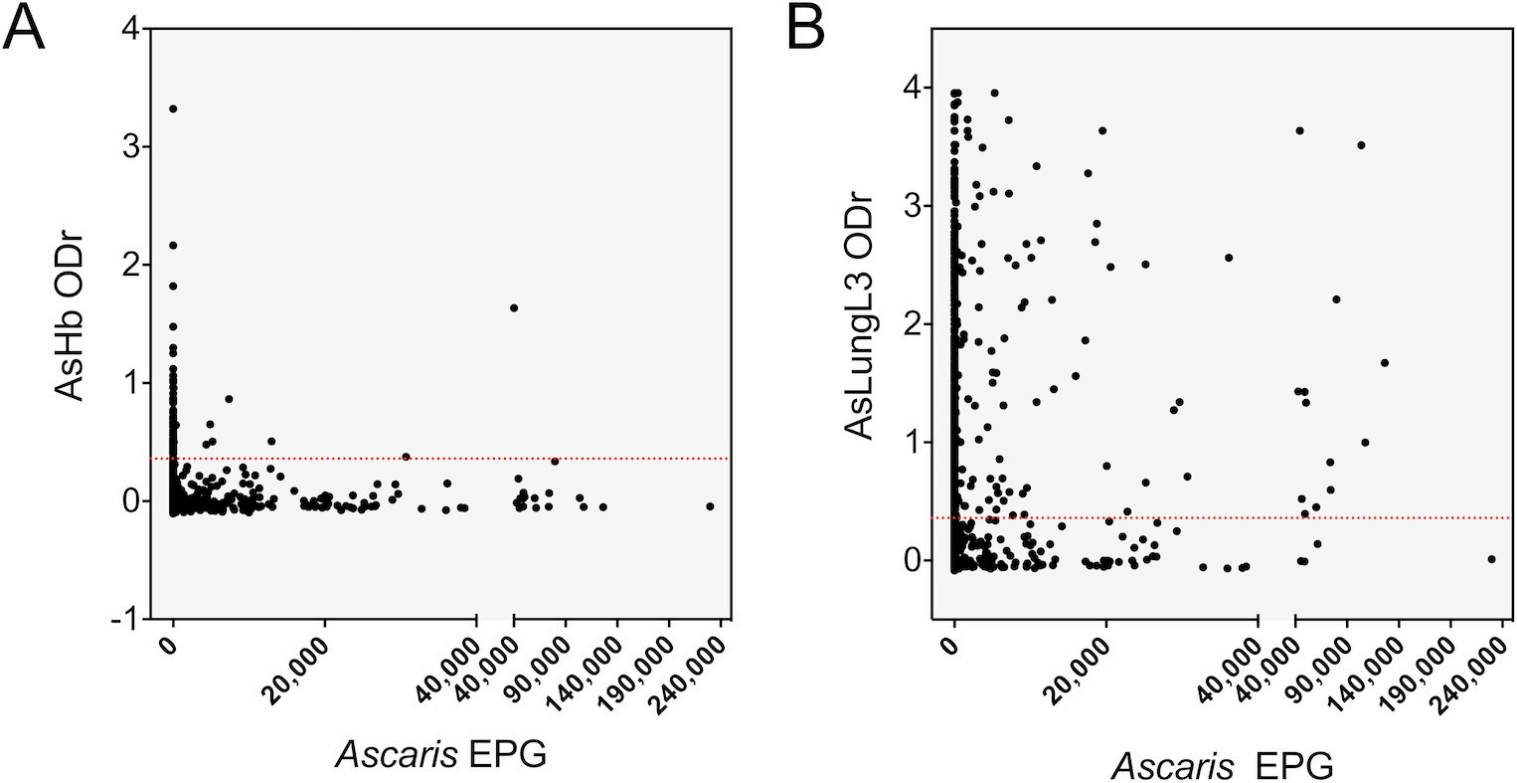
Relationship between *Ascaris* fecal egg counts and antibody response to *Ascaris* antigens. IgG4 antibodies to *Ascaris suum* haemoglobin (AsHb) (A) and lung L3 extract (AsLungL3) (B) measured in the 1,800 sampled SAC and adult individuals from Jimma Town, Ethiopia are plotted against fecal egg counts (FECs) obtained by Kato-Katz. FECs are expressed as eggs per gram of stool (EPG)). The horizontal red dotted lines represent the assay-specific cutoffs.

Using the complete dataset (surveys 1 to 3, n = 1,800), we investigated the relationship between the IgG4 antibody responses to the *Ascaris* antigens (ODr) and the presence and intensity of STH infections (FECs) (**Fig 5**). A large number of the individuals that excreted *Ascaris* eggs were seronegative (AsHb: 96.8% (306/316) and AsLungL3: 39.2% (124/316)). In contrast, many individuals who did not excrete any *Ascaris* eggs were seropositive (AsHb: 4.2% (63/1,484) and AsLungL3: 56.5% (838/1,484)). Individuals excreting *Ascaris* eggs did not show a higher antibody response to either *Ascaris* antigen (t-test: AsHb: *p* = 0.075 and AsLungL3: *p* = 0.083), nor did we observe a noteworthy relationship between the *Ascaris* FECs measured by Kato-Katz and the intensity of the IgG4 response to AsHb (Pearson’s coefficient = 0.03 *p* = 0.223) or AsLungL3 (Pearson’s coefficient = 0.05, *p* = 0.04).

### Assessment of cross-reactivity

**Fig 6:**
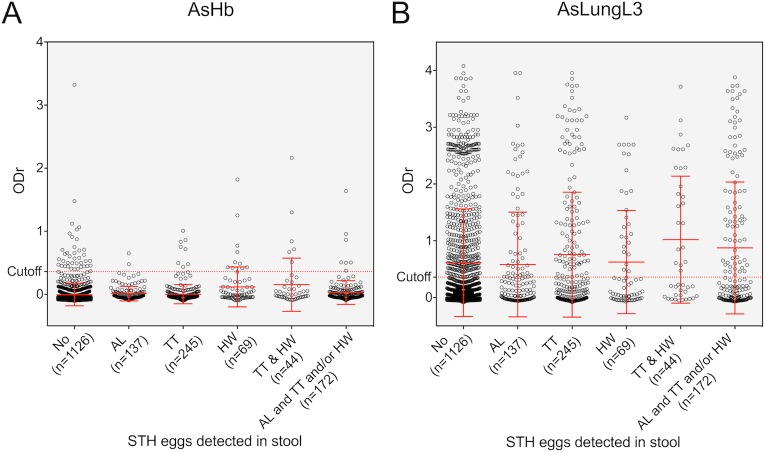
Impact of helminth coinfections on serological response to *A*. *suum* antigens. These plots show the IgG4 antibody reactivity to *A. suum* haemoglobin (AsHb) (A) and total lungL3 extract (AsLungL3) (B) in 1,200 SAC and 600 adults from Jimma Town, Ethiopia. Individuals are categorized by soil-transmitted helminth (STH) status. Group means and standard deviations are indicated in red (No = no STH eggs detected; AL: only positive for *Ascaris*; TT: only positive for *Trichuris*; HW: only positive for hookworms; TT&HW: both *Trichuris* and hookworm eggs detected in the stool; AL and TT and/or HW: *Ascaris* eggs detected by copromicroscopy together with *Trichuris* and /or hookworm eggs).

Using the complete dataset again (including all 1,800 samples), we investigated the effect of infection with other STHs on the serological IgG4 response to the *Ascaris* antigens (cross-reactivity). For this, individuals were grouped according to the STHs detected in their stool sample. Depending on the type of eggs detected in the stool during Kato-Katz examination, individuals were labeled as not infected, infected with *Ascaris*, *Trichuris* or hookworm only, having a mixed infection with *Trichuris* and hookworm or being infected with *Ascaris* plus at least one additional STH species. The serological responses of these individuals to AsHb and AsLungL3 is presented in **Fig 6**. Both graphs indicate that, independent of the presence or type of infection present in the individual at the time of sampling, average antibody responses to the *Ascaris* antigens are very similar.

## Discussion

The scientific community has recently summarized the desired characteristics for diagnostic tools across different phases of MDA programs. As substantiated by other NTD programs, serology holds promise as an alternative diagnostic tool when the program approaches the endgame [7, 33]. To supplement the scarcity of data on baseline serological profiles in human populations, we measured anti-*Ascaris* IgG4 antibodies in SAC and adults residing in both an STH endemic (Jimma Town, Ethiopia) and a non-endemic (Belgium) area using two different in-house ELISAs based on two *A*. *suum* antigen preparations. In addition, we compared the infection profiles in SAC before and after 3 years of biannual school-based MDA using copromicroscopy and serology.

### Adult population remains a reservoir for STH transmission

Although both coproprevalence and mean FECs dropped significantly as a function of increasing age, the adult population in Jimma Town still hosts a substantial reservoir of adult worms, capable of maintaining transmission in the population. Both deterministic and stochastic STH transmission models [5], as well as recent findings by Dunn et al. [34], who found that adults were major contributors to total STH prevalence and FECs, seem to underpin the notion that broadening coverage to encompass all age classes will likely be a more effective approach for the long-term control of STHs than restricting deworming to SAC only. However, at this moment, the impact of school-based MDA on the STH transmission in the adult population is not well known. More longitudinal monitoring and evaluation studies are needed to evaluate the long-term impact of these control strategies across the whole population.

### Increased antibody response against *Ascaris* antigens in some Belgian adults

In this study, the cutoff values for both ELISAs (AsHb and AsLungL3 ELISA) were determined using 500 serum samples from healthy adult blood donors in Belgium as negative population. Despite the absence of STH in Belgium, a number of serum samples did show increased antibody responses to AsHb and AsLungL3 antigens. There are a number of possible explanations for these findings, including zoonotic infections with canine or feline *Toxocara* spp. or the porcine helminth *Ascaris suum*. *Toxocara* spp. are closely related to *A*. *lumbricoides* and are prevalent in domesticated cats and dogs in Belgium [31, 32]. To investigate the presence of *Toxocara* infections, a set of 35 samples that showed elevated IgG4 responses to the *Ascaris* antigens and 30 samples that did not respond to either *Ascaris* antigens were screened on a commercial *Toxocara* ELISA. Only two *Ascaris* seropositive samples showed a positive result on the *Toxocara* ELISA, suggesting that there is limited cross reactivity between the respective ELISAs. The porcine roundworm *A. suum* is still highly prevalent in the Belgian pig industry [14, 16] and has been shown to infect humans [35–38]. We also cannot exclude that exposure to STHs or *Toxocara* species while travelling abroad could have occurred [39, 40], as we did not have any background information on our Belgian serum donors. These elevated antibody levels against *Ascaris* antigens in a number of Belgian samples does however confirm a serological study performed in the Netherlands that found 45 out of 629 (7.2%) children to be *Ascaris*-seropositive [41]. Yet, here too, it remains unknown what the source of infections is, and to what extent these infections have any health consequences. For future serological studies, the availability of sera from young, unexposed, non-endemic individuals would be of value for assay cut-off determination.

### Exposure to the infectious stages of *Ascaris* reaches beyond SAC

Anti-AsLungL3 seroprevalence was 45.0% in the youngest age group and steadily increased with age reaching nearly 90% seroprevalence in individuals over the age of 50. Such findings can be interpreted as evidence that individuals are continuously exposed to the larval stages of *Ascaris* from a very early age onwards, and that the absence of *Ascaris* eggs in the stool of older individuals does not necessarily imply the absence of exposure to larval stages. The fact that *Ascaris* exposure is of all ages is fostered by the fact that patent infections were detected in all age groups. The oldest individual found to excrete *A*. *lumbricoides* eggs was 85 years old.

### The AsLungL3 extract contains promising antigens for further diagnostic tool development

Serological analysis of the sera collected in this study revealed a significant difference in IgG4 antibody reactivity to the two *Ascaris* antigen preparations. Relatively few individuals had an elevated IgG4 response to AsHb, while many individuals showed elevated sero-reactivity to the AsLungL3 antigen. One reason for the low number of AsHb seropositive individuals could be due to the way the assay cut-off was determined. The cut-off for the AsHb assay was notably higher than the cut-off for the AsLungL3 ELISA. This is the direct consequence of the fact that more Belgian individuals had an elevated antibody response to the AsHb than to the AsLungL3 extract (**Fig 2B**), resulting in a significantly larger standard deviation around the average ODr, and thus a higher cut-off for AsHb assay (AsHb: 0.36 ODr *vs*. AsLungL3: 0.08 ODr). However, this fact alone clearly does not explain the large discrepancy between the results obtained by both ELISAs. Overall, there appears to be a much more pronounced IgG4 antibody response to the AsLungL3 antigen compared to the AsHb antigen. This could partly be described to the fact that the AsLungL3 antigen is a complete larval extract that contains a multitude of larval antigens and epitopes for antibodies to bind to, whereas, AsHb is a single purified native antigen with a limited number of antibody binding targets. These results are however in correspondence with the results of a recent evaluation of the AsLungL3 extract as diagnostic antigen to measure exposure in piglets experimentally infected with *A*. *suum*. This study also found that the antibody response to AsLungL3 developed faster and was more pronounced than the one directed to AsHb [13]. Hence, determining the antigenic constitution of the AsLungL3 extract is of particular interest for future diagnostic test development.

By grouping individuals according to the type of patent infection they carried (as verified by copromicroscopy), we wished to evaluate the presence of cross-reactive antibodies to the *A*. *suum* antigens. However, antibody responses to these antigens were very similar across all different groups and it was not possible to draw conclusions with regards to the species-specificity of the assays. Based on qPCR results from a recent survey in the Jimma area, we do however know that the prevalence of other parasitic species that might cause antibody cross-reactivity such as *Schistosoma mansoni*, *Strongyloides stercoralis* and *Taenia* spp in SAC is very low (3.5%, 3.5% and 2.7% respectively). Moreover, Jimma town is considered hypo-endemic for *Onchocerca volvulus* [42] and non-endemic for other filarial infections including *Mansonella perstans* [43], *Loa loa* [44] or *Wuchereria bancrofti* [45, 46].

### Three years of biannual MDA reduces STH prevalence and serological response to *Ascaris* antigens in SAC

This study also compared coprological and serological results collected from SAC at the start of the STH control program in 2015 with those collected in 2018, three years into the program. The effects of the control program were clearly visible. Overall STH infection had dropped from 57.7% to 28.0% (51.5% reduction), and so did the intensity of the infections (*Ascaris*: 48.2%, *Trichuris*: 67.3% and hookworm: 69.6% reduction). Interestingly, the control program also led to a significantly reduced IgG4 response to both AsHb and AsLungL3, resulting in less seropositive individuals (AsHb: 40.9% reduction; AsLungL3: 27.4% reduction) and significantly lower mean ODr levels measured in the SAC (AsHb; 44.2% reduction; AsLungL3: 38.2% reduction). These findings support the results presented in the study of Vlaminck and colleagues from 2016 [19] where anti-AsHb IgG4 levels were evaluated in an Indonesian community subjected to community wide MDA with albendazole for lymphatic filariasis and soil-transmitted helminthiasis. Even though, in that particular population, significantly higher number of AsHb seropositive samples were detected at baseline (67.7% in 2002), the seroprevalence significantly reduced during the course of the intervention (22.6% in 2004, 23.0% in 2006 and 14.3% in 2009). At the same time, average *A*. *lumbricoides* coproprevalence in the community reduced with 22.8% (from 38.2% in 2002 to 29.5% in 2009) while community egg output reduced with 81.6% (from 698 EPG in 2002 to 128 EPG in 2009).

### Absence of association between coprological and serological results

It is important to note that many of the examined individuals excreting *Ascaris* eggs were seronegative (96.2% using the AsHb and 43.6% using the AsLungL3 assay) and that 82.1% (AsHb) and 79.9% (AsLungL3) of seropositive individuals did not excrete any *Ascaris* eggs. Other studies that compared anti-*Ascaris* antibody responses to adult worm counts and FECs also reported the absence of a significant association between antibody titers and worm load or egg production [19, 47, 48]. Moreover, King *et al*. [47] also noted a negative association between total IgG responses to larval *A*. *lumbricoides* antigen and infection intensity in young children (5–11 years old). There is no real straightforward explanation for this seemingly inversed correlation. One possibility is that continued *Ascaris* exposure acts as an ongoing booster to maintain elevated protective immune responses, hereby preventing the development of new patent infections. This is supported by earlier reports that have shown that Th2-mediated responses play an important role in the age-dependent reduction of intestinal helminth infections in humans [49, 50].

### Conclusions

During *Ascaris* infection, antibodies are raised to immunogenic components produced by the worm and which are detected and processed by the immune system. Such antibodies are unique in their capacity to serve as a biomarker to identify individuals that have experienced exposure to this infection. Measuring infection-induced antibodies has proven an objective way to monitor a population’s exposure to multiple NTDs [12, 51–54]. This study clearly demonstrated the positive impact of the MDA program on STH infection prevalence in Jimma Town, Ethiopia and further underpins the idea that it is possible to evaluate the impact of control efforts by measuring the changes in antibody responses to STH antigens. Combining a number of antibody assays in a multiplex format would provide a great opportunity to integrate monitoring and surveillance of a number of infectious diseases [55]. In order to progress towards a species-specific antibody test for *Ascaris*, it would be of special interest to identify the specific immunodominant antigens present in the AsLungL3 extract. In addition, it is absolutely necessary to collect more data on how to interpret and apply serological results. We need a far better understanding of the serological profiles produced by populations with different backgrounds of exposure to *Ascaris* and STHs in general.

## Supporting information

S1 Table The prevalence and intensity of STH infections based on copromicroscopy in Jimma Town, 2015. The table summarizes the prevalence and intensity of *Ascaris lumbricoides*, *Trichuris trichiura* and hookworm infections based on copromicroscopy (Kato-Katz, Mini-FLOTAC and McMaster) across different age groups, both sexes and nine *kebeles* at the start of the national deworming program. The intensity of infection is measured by mean fecal egg counts (FECS; expressed in eggs per gram of stool (EPG)).
(DOCX)

S2 Table The prevalence and intensity of STH infections based on serology in Jimma Town, 2015. This table summarizes the prevalence and intensity of *Ascaris lumbricoides* infections based on serology across five different age groups, both sexes and nine *kebeles* at the start of the national deworming program in Jimma Town, Ethiopia. The intensity of infection is measured by mean optical density ratio (ODr).
(DOCX)

S1 Info The complete study dataset. (CSV)
