# Supplementary material for: Evaluation of copromicroscopy and serology to measure the exposure to Ascaris infections across age groups and to assess the impact of 3 years of biannual mass drug administration in Jimma Town, Ethiopia
Source: PLoS Negl Trop Dis. 2020 Apr 13;14(4):e0008037. doi: 10.1371/journal.pntd.0008037 (PMC7179930; doi:10.1371/journal.pntd.0008037)
Supplement: S1 Table — The table summarizes the prevalence and intensity of Ascaris lumbricoides, Trichuris trichiura and hookworm infections based on copromicroscopy (Kato-Katz, Mini-FLOTAC and McMaster) across different age groups, both sexes and nine kebeles at the start of the national deworming program. The intensity of infection is measured by mean fecal egg counts (FECS; expressed in eggs per gram of stool (EPG)). (DOCX) [file pntd.0008037.s001.docx]

**S1 Table: The prevalence and intensity of soil-transmitted helminth infections based on copromicroscopy in Jimma Town, 2015.** The table summarizes the prevalence and intensity of *Ascaris lumbricoides*, *Trichuris trichiura* and hookworm infections based on copromicroscopy (Kato-Katz, Mini-FLOTAC and McMaster) across different age groups, both sexes and nine *kebeles* at the start of the national deworming program. The intensity of infection is measured by mean fecal egg counts (FECS; expressed in eggs per gram of stool (EPG)).

|  | | N | *Ascaris* | |  | *Trichuris* | |  | Hookworm | |
| --- | --- | --- | --- | --- | --- | --- | --- | --- | --- | --- |
|  |  |  | Prevalence (%) | Mean FEC (EPG) |  | Prevalence (%) | Mean FEC (EPG) |  | Prevalence (%) | Mean FEC (EPG) |
| Age group (years) | | | | | | | | | | |
|  | 5 –10 | 300 | 39.7 | 2,524 |  | 50.3 | 185 |  | 10.7 | 17 |
|  | 14 – 17 | 263 | 31.6 | 1,377 |  | 53.6 | 123 |  | 22.4 | 42 |
|  | 18 – 29 | 310 | 12.6 | 708 |  | 33.5 | 58 |  | 18.1 | 34 |
|  | 30 – 49 | 208 | 11.1 | 646 |  | 28.4 | 83 |  | 5.3 | 18 |
|  | ≥50 | 119 | 3.4 | 129 |  | 25.2 | 58 |  | 6.7 | 11 |
|  |  |  |  |  |  |  |  |  |  |  |
| Sex | | | | | | | | | | |
|  | Male | 496 | 27.2 | 1,168 |  | 44.8 | 125 |  | 16.7 | 28 |
|  | Female | 704 | 18.9 | 1,291 |  | 37.4 | 96 |  | 11.8 | 25 |
|  |  |  |  |  |  |  |  |  |  |  |
| Kebele | | | | | | | | | | |
|  | Awetu Mendera | 60 | 43.3 | 2,269 |  | 50.0 | 201 |  | 11.7 | 68 |
|  | Bacho Bore | 160 | 23.8 | 1,318 |  | 43.8 | 107 |  | 12.5 | 22 |
|  | Bossa Addis | 160 | 18.8 | 867 |  | 38.1 | 53 |  | 22.5 | 21 |
|  | Bossa Kitto | 160 | 11.2 | 1,599 |  | 31.2 | 136 |  | 13.1 | 50 |
|  | Ginjo | 220 | 25.5 | 1,299 |  | 42.3 | 99 |  | 11.4 | 16 |
|  | Hermata | 60 | 31.7 | 1,620 |  | 43.3 | 158 |  | 15.0 | 32 |
|  | Jiren | 60 | 36.7 | 1,598 |  | 48.3 | 89 |  | 20.0 | 17 |
|  | Mentina | 160 | 18.1 | 1,129 |  | 44.4 | 165 |  | 6.9 | 24 |
|  | Seto Semaro | 160 | 18.8 | 546 |  | 34.4 | 46 |  | 15.6 | 14 |
|  | **Total** | **1,200** | **22.3** | **1,240** |  | **40.4** | **108** |  | **13.8** | **26** |
