# Supplementary material for: Evaluation of copromicroscopy and serology to measure the exposure to Ascaris infections across age groups and to assess the impact of 3 years of biannual mass drug administration in Jimma Town, Ethiopia
Source: PLoS Negl Trop Dis. 2020 Apr 13;14(4):e0008037. doi: 10.1371/journal.pntd.0008037 (PMC7179930; doi:10.1371/journal.pntd.0008037)
Supplement: S2 Table — This table summarizes the prevalence and intensity of Ascaris lumbricoides infections based on serology across five different age groups, both sexes and nine kebeles at the start of the national deworming program in Jimma Town, Ethiopia. The intensity of infection is measured by mean optical density ratio (ODr). (DOCX) [file pntd.0008037.s002.docx]

**S2 Table: The prevalence and intensity of soil-transmitted helminth infections based on serology in Jimma Town, 2015.** This table summarizes the prevalence and intensity of *Ascaris lumbricoides* infections based on serology across five different age groups, both sexes and nine *kebeles* at the start of the national deworming program in Jimma Town, Ethiopia. The intensity of infection is measured by mean optical density ratio (ODr).

|  | | **N** | **AsHb ELISA** | |  | **AsLungL3 ELISA** | |
| --- | --- | --- | --- | --- | --- | --- | --- |
|  |  |  | Prevalence (%) | Mean ODr |  | Prevalence (%) | Mean ODr |
| **Age group (in years)** | |  |  |  |  |  |  |
|  | 5 –10 | 300 | 3.3 | 0.016 |  | 45.0 | 0.445 |
|  | 14 – 17 | 263 | 3.8 | 0.029 |  | 63.1 | 0.761 |
|  | 18 – 29 | 310 | 5.8 | 0.008 |  | 71.0 | 0.833 |
|  | 30 – 49 | 208 | 6.7 | 0.034 |  | 80.3 | 1.147 |
|  | ≥50 | 119 | 6.7 | 0.015 |  | 89.1 | 1.634 |
|  |  |  |  |  |  |  |  |
| **Sex** | |  |  |  |  |  |  |
|  | Male | 496 | 5.6 | 0.040 |  | 66.5 | 0.817 |
|  | Female | 704 | 4.5 | 0.006 |  | 65.9 | 0.881 |
|  |  |  |  |  |  |  |  |
| **Kebele** | |  |  |  |  |  |  |
|  | Awetu Mendera | 60 | 3.3 | 0.021 |  | 43.3 | 0.542 |
|  | Bacho Bore | 160 | 7.5 | 0.019 |  | 61.2 | 0.795 |
|  | Bossa Addis | 160 | 7.5 | 0.050 |  | 68.1 | 0.832 |
|  | Bossa Kitto | 160 | 4.4 | 0.004 |  | 70.0 | 0.909 |
|  | Ginjo | 220 | 2.7 | 0.000 |  | 69.1 | 1.039 |
|  | Hermata | 60 | 1.7 | -0.008 |  | 66.7 | 0.582 |
|  | Jiren | 60 | 3.3 | 0.021 |  | 43.3 | 0.418 |
|  | Mentina | 160 | 7.5 | 0.049 |  | 75.0 | 1.063 |
|  | Seto Semaro | 160 | 3.8 | 0.013 |  | 69.4 | 0.801 |
|  | **Total** | **1,200** | **5.0** | **0.020** |  | **66.2** | **0.854** |
